# Managing Worrying About Worrying with Metacognitive Restructuring Versus Metacognitive Defusion

**DOI:** 10.3390/bs16040594

**Published:** 2026-04-16

**Authors:** Robert D. Zettle, Huan Quan, Jonathan M. Larson

**Affiliations:** Department of Psychology, Wichita State University, 1845 Fairmount, Wichita, KS 67260, USAjmlarson1@shockers.wichita.edu (J.M.L.)

**Keywords:** generalized anxiety disorder, metacognitive restructuring, metacognitive defusion, process-based cognitive behavioral therapy

## Abstract

Process-based cognitive behavioral therapy (PB-CBT) seeks to identify kernels that are equally efficacious in treating various disorders. While both metacognitive therapy (MCT) and acceptance and commitment therapy (ACT) represent evidence-based interventions for generalized anxiety disorder (GAD), it remains unclear if there are specific components within each that are comparable in managing meta-worrying characteristic of GAD. A subclinical sample of college students received a brief session targeting a single, personally relevant meta-worry of either metacognitive restructuring as practiced in MCT or an extension of ACT defusion exercises. Both were equally effective in impacting believability, distress, and willingness ratings of the targeted worry as well as untargeted worries. However, significantly longer metacognitive defusion sessions suggest that metacognitive restructuring may represent a more cost-effective option. Levels of generalized anxiety, dispositional worrying, and depression did not moderate treatment responsivity. Implications of findings for PB-CBT research and practice are discussed within the context of study limitations.

## 1. Managing Worrying About Worrying with Metacognitive Restructuring Versus Metacognitive Defusion

Generalized anxiety disorder (GAD) is often a chronic and potentially debilitating form of emotional suffering with somewhat varying reports of its epidemiology (Druet-Cabanac et al., 2025). Estimates of GAD’s 12-month prevalence rates among U.S. adults have ranged from 3.1% (Anxiety and Depression Association of America, n.d.) to 7.4% (Druet-Cabanac et al., 2025), while generally lower figures have been reported for other parts of the world, particularly among less economically developed countries (Ruscio et al., 2017). The current diagnostic and statistical manual (American Psychiatric Association, 2022) as well as international classification of diseases (World Health Organization, 2022) concur that an essential and defining feature of GAD is excessive worry, which in turn is associated with physical symptoms, such as fatigue and muscle tension, and resulting clinically significant distress or impairment in one or more areas of functioning.

What have come to be regarded as traditional forms of cognitive behavioral therapy (tCBT; Fang & Ding, 2023) have been widely recognized as having the strongest empirical support in treatment of GAD (Society of Clinical Psychology, n.d.). Such interventions representative of CBT’s second wave (Hayes, 2004) featured efforts to dispute and cognitively restructure worrying that was largely if not exclusively centered on external events and related concerns (e.g., “If I go somewhere unfamiliar to me, I’ll get robbed”), or what proponents of metacognitive therapy (MCT; Wells, 2009) have referred to as Type 1 worries. Of particular relevance to this project is that the metacognitive model of GAD also implicates Type 2 or meta-worries that reflect worrying about worrying. While recent research found that both types of worrying significantly account for unique variance in symptoms of GAD, Type 2 worries were more influential (Nordahl et al., 2023), thus underscoring the importance of identifying treatment packages and/or components within them that successfully weaken the impact of meta-worrying.

Presently, how the efficacy of MCT as well as more recent variants of CBT for GAD, such as acceptance and commitment therapy (ACT; Hayes et al., 2012), compare to tCBT and to each other remains unclear. The Society of Clinical Psychology (n.d.) has concluded that “there is not yet sufficient evidence to list these approaches as empirically-supported” despite at least some findings that might suggest otherwise. For example, a recent meta-analysis reported that GAD clients treated with MCT attained greater reductions in levels of worrying and anxiety at both posttreatment and follow-up compared to those receiving tCBT (Rawat et al., 2023). In particular, GAD-related recovery rates were higher at 9-years posttreatment for those treated with MCT versus tCBT (Solem et al., 2021). While ACT for GAD has not been shown to be superior to tCBT (Twohig et al., 2024), it has been found to be just as efficacious in providing symptomatic relief (Stefan et al., 2019) and more impactful than MCT in enhancing psychological well-being (Mostafazadeh et al., 2024).

A more process-based approach to CBT (PB-CBT; Hayes & Hofmann, 2018; Hofmann & Hayes, 2019; Svitak & Hofmann, 2024) has recently been offered as an alternative to comparing branded CBT packages for specific diagnostic subgroups to each other in a series of randomized clinical trials (e.g., Task Force, 1995). For example, tCBT and ACT have been found to be equally efficacious in treating anxiety disorders more broadly (Arch et al., 2012) and GAD in particular (Dinarvand et al., 2022). In contrast to such a “protocols for syndromes” approach (Hayes et al., 2019) that has been used for the last three decades to determine the relative levels of empirical support for specific therapeutic protocols and packages, PB-CBT instead focuses on identifying shared mechanisms of change that can be linked with evidence-based therapeutic components or kernels (Embry & Biglan, 2008) that reliably move such processes. Instead of a nomothetic, one-size-fits-all approach in which protocols are selected from among those that are empirically supported for clients with a shared diagnosis, PB-CBT seeks to personalize individual therapy by identifying and targeting for change key processes uniquely contributing to each client’s presenting concerns (Moskow et al., 2023). In doing so, PB-CBT expands upon other, albeit more limited transdiagnostic approaches, such as the unified protocol (UP; Barlow et al., 2011), that have also incorporated multiple treatment components linked at least conceptually to central processes. However, while UP is limited to emotional disorders, PB-CBT seeks to be broader in its transdiagnostic reach, and also to be more comprehensive in its inclusion of kernels than the 5–8 modules of UP. Consistent with the agenda of PB-CBT, the major purpose of this project was to compare the impact of therapeutic elements integral to MCT (cognitive restructuring) and ACT (defusion) in treating GAD within an analog preparation.

An example relevant to the ultimate objective of this project and that of PB-CBT more broadly would be ascertaining optimally effective ways of targeting worries, provided that successfully doing so, in turn, can be shown to mediate desired therapeutic outcomes, including symptomatic relief in addition to enhanced quality of life, among those who struggle with GAD. Evidence that the purported mechanism of treatment (i.e., reducing the influence of worrying) is closely related to GAD’s suspected mechanism of disorder (i.e., the dominance and pernicious consequences of worrying) would further support the strategic mission of PB-CBT (David & Montgomery, 2011). If its agenda is successfully met, PB-CBT has the possibility of affording clinicians increased therapeutic flexibility. Rather than being rigidly guided by packaged treatment manuals and protocols organized around diagnostic categories, CBT practitioners could, for example, potentially successfully target the same transdiagnostic process shared by a range of psychological disorders with an array of techniques and procedures that have been shown to predictably move it.

A consideration of both similarities and differences in how tCBT, MCT, and ACT address worrying as a defining feature of GAD may help inform a PB-CBT approach to treating it. Particularly more cognitively oriented forms of tCBT and MCT both in effect seek to restructure salient worries in GAD through the use of Socratic questioning, disputation, and the gathering and processing of disconfirming evidence via in-session exercises and behavioral homework assignments. They differ from each other, however, primarily in the types and levels of worrying being targeted. While tCBT focuses on changing the content of anxiety-eliciting automatic or Type 1 thoughts (e.g., “Someone will break into my house”) and related supporting dysfunctional beliefs (e.g., “The world is a very dangerous and unpredictable place”), MCT addresses Type 2 cognitions or meta-worries about worrying itself such as “My worrying is dangerous for me” (Wells, 2009).

ACT, like tCBT, also primarily focuses on automatic Type 1 thoughts related to GAD, but through the use of cognitive defusion rather than restructuring techniques. Such techniques within ACT, such as word repetition (e.g., rapidly repeating aloud a triggering word like “dangerous”) and thought prefacing (e.g., “I have the thought that traveling alone is dangerous”) are typically presented as experiential exercises (Blackledge, 2015). Their immediate intended purpose is to create some distance or disentanglement from worries rather than alter their content, thereby enabling clients to relate to such concerns as mere psychological experiences rather than as immutable facts. Within the model of human functioning on which ACT is based, defusion is posited as one of six processes that contribute to psychological flexibility or the ability to successfully pursue valued goals in the face of emotional barriers such as generalized anxiety (Hayes et al., 2012). Consequently, the successful application of defusion techniques should also contribute to enhanced psychological flexibility.

This comparative overview of tCBT, MBC, and ACT suggests at least two options within PB-CBT for successfully treating GAD. One is to target Type 1 worries with either cognitive restructuring procedures from tCBt or cognitive defusion exercises from ACT. The other option is to address Type 2 meta-worries with metacognitive restructuring methods from MCT or by extending the application of defusion techniques to them. Unfortunately, because randomized clinical trials typically have compared one treatment package or protocol against another, it often remains unclear what specific and possibly unique elements within each account for the overall findings. One rather effortful, but ostensibly more definitive means of clarifying this ambiguity is to conduct a component analysis with clinical samples (e.g., Borkovec et al., 2002). An alternative and more expeditious strategy like that employed in this project is to conduct laboratory-based analog studies in which isolated and well-defined CBT-based procedures and techniques are presented to nonclinical and/or subclinical samples (e.g., Levin et al., 2012).

To reiterate, the primary purpose of this project was to evaluate how defusion procedures may compare with MCT techniques in targeting meta-worries. The previously cited comparison of ACT and MCT (Mostafazadeh et al., 2024) does not adequately speak to this question and we are unaware of any other research that does so that could be used to help guide the design and execution of this study. Rather, we primarily relied on the results of another recent project of ours (Quan & Zettle, 2023) and previous analog investigations comparing defusion and cognitive restructuring ala tCBT in targeting distressing automatic thoughts. Among a convenience sample of college students, negative beliefs about worry and cognitive fusion both independently accounted for significant variability in levels of generalized anxiety (Quan & Zettle, 2023), suggesting that targeting meta-worries with defusion kernels may be another viable therapeutic option.

To our knowledge, this study is the first to compare what might be termed as metacognitive defusion with metacognitive elements from MCT for addressing worrying about worrying in the laboratory. There have, however, been at least two analog studies that compared brief presentations of defusion to cognitive restructuring as practiced in tCBT in targeting negative self-referential thoughts (Deacon et al., 2011; Larsson et al., 2016) that helped inform the methodology and design of this project. As in the Deacon et al. (2011) study, we also did not include a control condition in light of previous research documenting the salutary effects of both cognitive defusion (Levin et al., 2012) and metacognitive techniques (Capobianco & Nordahl, 2023) when evaluated separately from each other. Rather, our prescreened college student participants were randomly assigned to receive a brief (15–20 min) presentation of one of our two interventions targeting a personally relevant meta-worry. Like (Larsson et al., 2016), we included a measure of willingness to have the targeted as well as related, but untargeted, negative beliefs about worrying in addition to analyses of associated distress and believability in comparing the two presentations. By including both targeted and untargeted meta-worries we were able to evaluate the degree to which the impact our of two interventions in effect generalized to “untreated” worries about worries.

While we had some reason to expect that defusion procedures and exercises might be equally beneficial in both decreasing distress and believability and increasing acceptance, as reflected by the willingness to have meta-worries, as has been shown to be the case with negative automatic thoughts, this expectation was tempered by the lack of any research that has extended such techniques to Type 2 worries. Accordingly, it seemed more probable that any difference between our two interventions would favor metacognitive restructuring given its prominent role within MCT. Equivalent outcomes, however, would suggest that clinicians might have two viable options—both metacognitive defusion and metacognitve restructuring—to assist those who struggle with the meta-worries that typify GAD.

Such findings by themselves would ostensibly provide additional support for PB-CBT writ large, but could be further supplemented by also examining at least a few variables that for coherent theoretical and conceptual reasons might be expected to function as possible moderators of intervention outcomes (Hayes & Hofmann, 2018, Chapter 29). Unfortunately, knowing at the aggregate level that those who struggle with GAD benefit equally from two treatment components does not inform clinicians what specific client characteristics may be associated with optimal outcomes at the level of individuals, regardless of which intervention is chosen (simple moderation), or those that may vary depending on the selected option (moderated moderation). To address this matter, in an exploratory manner we also examined as putative moderating variables levels of generalized anxiety and worry itself, as well as depression given its high rates of comorbidity with GAD among outpatient samples (Druet-Cabanac et al., 2025).

## 2. Method

### 2.1. Participants

**Figure 1:**
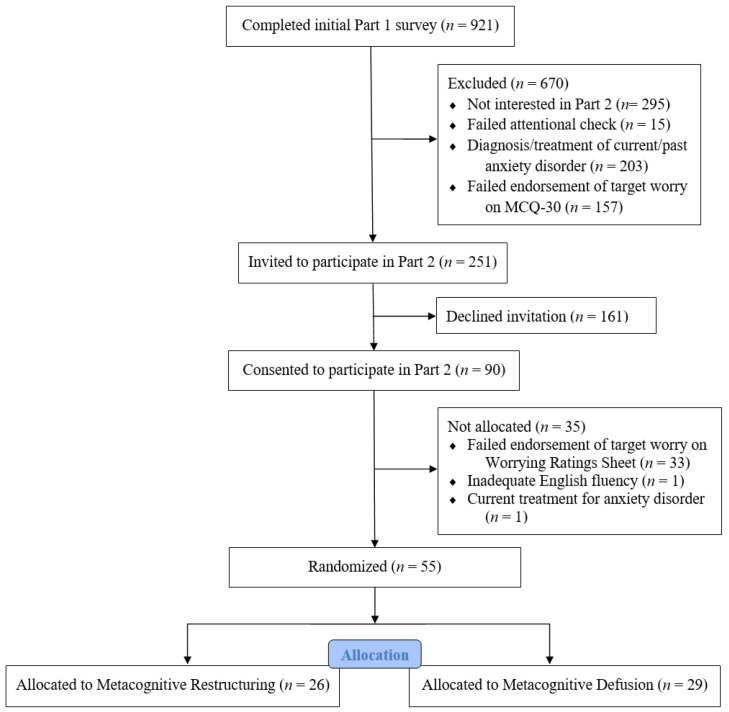
CONSORT flowchart of participants.

This two-part project was approved by an institutional review board and conducted in accordance with the ethical principles of the American Psychological Association (2017). During the initial screening or pretreatment phase of the study, an invitation to participate in Managing Your Worries—Part 1, described as “an anonymous online survey to assess your experiences with worrying, some of your beliefs about it, and how worrying may impact your life,” was posted on the Sona System used for the recruitment of psychological research participants. From July 2021 to December 2024, a fully completed survey was obtained during time periods in which it was active from college students (*N* = 921) whose participation was one means of fulfilling an introductory psychology course requirement. As indicated in Figure 1, at the end of the survey, 626 (68%) indicated an interest in participating in the second part of the study if deemed eligible and accordingly provided a contact email address. Students were excluded from further participation in this treatment phase of the project for one or more of the following reasons: (a) failure to pass an attentional check (*n* = 15), (b) a diagnosis of and/or current/past treatment of an anxiety disorder (*n* = 203), and (c) failure to indicate at least “a lot” of related distress and moderate agreement with one or more items that load on the negative beliefs about worry subscale of the Metacognitions Questionnaire-30 (MCQ-30; Wells & Cartwright-Hatton, 2004) (*n* = 157). In short, we sought to select participants experiencing an elevated level of distress related to meta-worrying, while also screening out those who might have received MCT and/or ACT as part of their treatment history.

The remaining 251 students who met all the inclusion criteria for phase two were emailed a password to schedule a timeslot for participation in Managing Your Worries—Part 2, described as providing “an opportunity to apply a psychological approach for better dealing with worrying,” with 90 (36%) doing so. Of this number, 33 were dismissed before receiving one of the interventions for failure to identify a targeted meta-worry at both a sufficiently high level of agreement and related distress, 1 for displaying inadequate English fluency, and another who reported initiating treatment for an anxiety disorder since completion of the screening survey (see Figure 1). The final retained sample (*N* = 55) was predominately female based on both birth gender as well as current gender identity (73%), non-Latine (80%), and White (70%) with a mean age of 20.13 years (*SD* = 4.61). As indicated in Table 1, participants who self-identified as Asian (12%), Black (11%), and Native American (7%) represented other racial subgroups.

### 2.2. Measures

***Demographic and Background Information Questionnaire***

This inventory was completed twice by participants—first, as part of the screening survey and again at the start of the treatment phase—to verify demographic information and any past diagnoses of anxiety disorder as well as related previous and/or current treatment.

***Pretreatment Screening Survey Measures***

Additional measures collected at pretreatment during the screening phase of this project were either used to further determine the eligibility of students to participate in the treatment phase or were subsequently analyzed as possible moderating variables of responsivity to the two interventions.

**Metacognitions Questionnaire-30 (MCQ-30).** The MCQ-30 is a self-report inventory of meta-beliefs typically addressed within MCT (Wells, 2009). We modified it in three respects to screen for participants who reported being sufficiently distressed by at least one moderately endorsed Type 2 worry. First, we administered only the six items that load on the negative beliefs about worry subscale (e.g., “my worrying is dangerous for me”). Second, in order to increase the range of endorsements, we added another response option (i.e., 3 = *neither agree nor disagree*) to the normal 4-point Likert scale (1 = *do not agree* to 4 *= agree very much*) used to assess belief in each of the subscale items (Wells & Cartwright-Hatton, 2004). The resulting scale assessing degree of agreement with negative beliefs about worrying displayed a questionable level of internal consistency (α = 0.63) that falls below those reported for the unmodified subscale by others (Wells & Cartwright-Hatton, 2004; Quan & Zettle, 2023). Lastly, we asked participants to also separately rate the level of distress caused by each of the six meta-worries according to a 5-point Likert scale (1 = *none at all* to 5 = *a great deal*), which when summed created a scale reflecting distress over meta-worries with an acceptable level of reliability (α = .71).

**Generalized Anxiety Disorder Scale (GAD-7).** The GAD-7 (Spitzer et al., 2006) is commonly used to screen for GAD in health care practice (Plummer et al., 2016; Sapra et al., 2020) and to assess the severity of its symptoms in clinical research (Toussaint et al., 2020). The seven items of the scale (e.g., “not being able to stop or control worrying” and “trouble relaxing”) are rated on a 4-point Likert scale (0 = *not at all* to 3 = *nearly every day*), with elevated levels of generalized anxiety indicated by higher scores. GAD-7 has demonstrated high levels of internal (α = .81 in this study) and test–retest reliability, in addition to satisfactory levels of criterion and construct validity (Spitzer et al., 2006). Research with both general population (Villarreal-Zegarra et al., 2024) and clinical samples (Johnson et al., 2019) has provided further support for the overall psychometric properties of the measure.

**Penn State Worry Questionnaire (PSWQ).** The PSWQ (Meyer et al., 1990) is designed to assess dispositional worrying with 16 items (e.g., “my worries overwhelm me” and “I am always worrying about something”) rated on a 5-point Likert scale (1 = *not at all typical of me* to 5 = *very typical of me*). Research with varying clinical populations (Brown et al., 1992; Wilson et al., 2025) has documented acceptable to good levels of the instrument’s temporal and internal consistency (α = .89 in this project) as well as support for its discriminant, convergent, and concurrent validity.

**Beck Depression Inventory-II (BDI-II).** The BDI-II (Beck et al., 1996) is widely used to assess the self-reported severity of symptoms of depression in both clinical and nonclinical samples (Wang & Gorenstein, 2013). Its 21 items are rated on a 4-point (0 to 3) Likert scale with higher total scores, ranging from 0 to 63, reflective of more severe levels of depression. Its internal (α = .90 in this study) and test–retest reliability as well as various types of validity (e.g., discriminant, concurrent, convergent, etc.) by now have been well-established with a wide range of global populations (e.g., Biracyaza et al., 2021; Hasan & Khan, 2025).

***Worrying Ratings Sheet***

Participants provided separate ratings during the treatment phase for each of the six items that load on the negative beliefs about worry subscale of the MCQ-30 in response to the following three questions: (a) “How much do you believe this to be true?” (0 = *not at all* to 100 *very much*), (b) “How much distress does this belief cause you?” (0 = *no distress* to 100 *very much*), and (c) as an indicator of acceptance, “How willing are you to have this concern?” (0 = *not willing* to 100 *very willing*). For each participant, one of the meta-worries was identified for therapeutic targeting with the three ratings for it analyzed separately from the mean ratings of believability, distress, and willingness for the five untargeted worries.

### 2.3. Procedure

Those who accepted an invitation to participate in the treatment phase of this project were randomly assigned to one of the two metacognitive interventions. After providing informed consent, participants completed the same demographic and background questionnaire included in the screening survey to ensure that all eligibility criteria were still being met. As previously mentioned, one participant who initiated treatment for anxiety since completion of the survey was dismissed at this point for this reason.

Participants next completed the Worrying Ratings Sheet in order to identify a personally relevant meta-worry for targeting by the interventions. To be eligible, a meta-worry had to be rated ≥60 on both believability and distress as well as ≤40 on willingness. As noted earlier, 33 participants for whom such a targeted worry could not be identified at this preintervention stage were dismissed. For participants with more than one eligible worry, the one judged as most problematic based on the combination of the three ratings was selected as the target. Of the six possible meta-worries, “When I start worrying, I cannot stop” was the most commonly identified targeted belief for 17 of 55 (31%) participants. Ratings of the five untargeted worries were summed together and the means analyzed separately from those of the targeted worry.

***Features Common to Both Interventions***

Both interventions were designed to be no longer than 30 min and were delivered in person by advanced doctoral students with more experience and training in ACT than MCT guided by protocols for each.^1^ Apart from two administrative oversights, the duration of each session was noted and the majority (31 of 55) were audio recorded, with 27 of them later reviewed as part of a check on treatment integrity. Each intervention began with a shared rationale to normalize worrying by suggesting that “worrying is actually quite normal at least in a statistical sense in that the vast majority of us may do it more than we like” before deviating from each other for the remainder of their presentations. At the conclusion of each intervention and prior to debriefing, participants were re-administered the Worrying Rating Sheet in order to assess its impact on the targeted worry and the degree to which any benefits may have generalized in the aggregate to the five untargeted meta-worries.

**Metacognitive restructuring.** As part of the more specific therapeutic rationale for this condition, participants were informed that “one of the more effective ways of managing worries is to focus on ways to reduce unfounded concerns about our inability to control worrying and how it might possibly harm us.” Techniques and procedures incorporated within this protocol were informed by and adapted from the coverage of MCT for GAD by Wells (2009, Chapter 6) and were presented within the context of an ongoing Socratic dialog with participants. Regardless of the specific targeted worry, therapeutic components to somewhat varying degrees emphasized (a) reviewing evidence and counterevidence for the targeted worry, (b) conducting in-session experimental tests of the targeted worry, such as those involving the uncontrollability of worrying (Wells, 2009, p. 109), and (c) questioning the mechanisms of harmful consequences attributed to the targeted worry (e.g., “If worrying is so dangerous, why is it never listed as a leading cause of death?”). As much as possible, tailored adaptations of these more general strategies and techniques within the protocol were made for each participant, depending on which of the six meta-worries was being targeted. For example, participants with a targeted worry of “I cannot ignore my worrying thoughts” were asked to reflect on whether their attention to them was disrupted during their completion of a brief in-session, anagram-solving task.

**Metacognitive defusion.** Participants were informed that “how much worrying we all do is less detrimental to our overall health and well-being than how we react to what our minds tell us about our worrying” to provide a rationale for a “focus on ways to disentangle ourselves from scary thoughts our minds tell us about worrying.” All participants completed an array of experiential defusion exercises focused on their targeted worry selected from a list of such interventions compiled by Strosahl et al. (2004) that included word repetition and/or thought prefacing within each session. In word repetition, a key emotionally eliciting word or phrase contained within articulation of the targeted worry (e.g., “dangerous”) was rapidly repeated aloud for up to 30 s (Masuda et al., 2009), while in thought prefacing, stating the entire worry aloud at a normal pace was preceded by first saying “I have the thought that …” (e.g., “I have the thought that my worrying is dangerous to me”). Additional defusion exercises presented to participants varied somewhat depending on their specific targeted worry and their responsivity to them. They included, but were not necessarily limited to, vocalizing the targeted worry very slowly, in song, or in a cartoon voice, all with the intended purpose of enabling participants to respond to their targeted worry as mere words rather than a factual assertion.

### 2.4. Data Analysis

We used IBM SPSS Statistics (Version 29) in conducting inferential statistical analyses to determine the degree to which pre to posttreatment changes in our dependent variables (i.e., believability, distress, and willingness ratings of targeted and untargeted meta-worries) could be attributed to our independent and/or moderating variables.

## 3. Results

### 3.1. Pretreatment Group Comparisons

We first compared our two groups based on data collected during the pretreatment screening survey in order to determine if the subsequent random assignment of participants to treatment conditions had the intended effect of creating two equivalent groups (see Table 1). No significant differences were noted on any of the demographic variables nor on any of the possible moderating variables with the exception that defusion group participants during the survey reported a higher level of collective distress associated with the six meta-worries that load on the negative beliefs about worry subscale of the MCQ-30, *t*(53) = 2.39, *p* = .02. We considered including this measure as a covariate or an additional possible moderator in subsequent analyses of treatment effects, but opted not to after determining that it was not correlated with changes in any ratings of either targeted or untargeted meta-worries for either treatment group.

### 3.2. Status of Putative Moderating Variables

An examination of the other possible moderating variables reported in Table 1 suggests that our participants in the aggregate were experiencing at least subclinical levels of comorbid GAD and depression. The mean GAD-7 score for our total sample (*M* = 10.29) falls within the moderate range (Sapra et al., 2020). The level exceeds that (*M* = 8.05) previously reported by a general student sample at our university (Quan & Zettle, 2023), *t*(280) = 2.82, *p* < .01, which may reflect a participant self-selection process given that this phase of the project, as previously mentioned, was advertised as “an opportunity to apply a psychological approach for better dealing with worrying.” The level of dispositional worrying as assessed by PSWQ *(M* = 62.46) appears to be even more elevated as it falls within the high range (Gillis et al., 1995). Overall, our sample also reported being mildly depressed based on a mean BDI-II score of 18.36 (Beck et al., 1996).

Among these three measures of emotional distress, only screening levels of generalized anxiety were associated with changes in any of the dependent variables. More specifically, for the total sample, GAD-7 scores correlated significantly with reductions in believability (*r* = .31, *p* = .021) and distress (*r* = .27, *p* = .047) involving untargeted worries, suggesting that improvement in both may have been moderated by levels of generalized anxiety. However, while GAD-7 scores also correlated with both ratings at pretreatment (believability *r* = .44, *p* < .001; distress *r* = .35, *p* < .01), they were unrelated to either at posttreatment (*r* = .14 and .11, respectively), indicating that greater reductions in believability and distress of untargeted worries for participants with higher levels of anxiety were instead attributable to pretreatment elevations in each. In effect, more anxious participants had greater room for improvement than their less anxious peers.

### 3.3. Treatment Integrity

Audio recordings of approximately half of the treatment sessions (27 of 55 = 49%) were independently reviewed by pairs of blind evaluators familiar with both protocols as a check on treatment integrity. For all, the evaluators were in complete agreement in correctly identifying which protocol was being presented.

In all but two instances, the duration of a treatment session was tracked by the graduate student therapist who conducted it, and if necessary, verified by reviewing the length of its recording. All sessions were concluded within the goal of 30 min or less (*M* = 20 min; range 12 min 7 s to 28 min), but with a significant difference noted between the two interventions, *t*(53) = 3.69, *p* < .001. The average defusion session (*M* = 21 min 40 s) was over 3.5 min longer than the typical restructuring presentation (*M* = 18 min 8 s). Because session duration for the entire sample was inversely correlated with pre- to posttreatment reductions in believability of targeted worries (*r* = −.34, *p* = .013) as well as increases in willingness to have untargeted worries (*r* = −.38, *p* < .01), we opted to include session duration as a covariate in the analysis of both measures.

### 3.4. Treatment Comparisons

Similar to other studies that have conducted laboratory comparisons of cognitive restructuring and defusion (Deacon et al., 2011; Larsson et al., 2016), we opted to conduct separate analyses for each of the three dimensions (e.g., believability, distress, and willingness) of both targeted and untargeted meta-worries. Related descriptive statistics for the six dependent variables are presented in Table 2, with no significant differences noted at pretreatment between the two conditions. Given the requirements of selecting a targeted worry for each participant, the pretreatment ratings of believability and distress for the total sample as well as the two treatment groups, as expected, were significantly higher and lower for willingness ratings (*p* < .01) than those for the untargeted worries.

The results of a series of 2 (Assessment occasion = pre vs. posttreatment) × 2 (Treatment condition = metacognitive restructuring vs. metacognitive defusion) mixed model analyses are summarized in Table 3. We evaluated ratings for targeted worry believability and willingness to have untargeted worries with ANCOVAs, with session duration as the covariate, while we conducted ANOVAs on the other four dependent measures. Significant improvements from pre- to posttreatment with medium to large effect sizes were noted for all six ratings that did not interact with treatment condition. The only other significant finding was a main effect for treatment condition on believability in the targeted worry, with restructuring participants reporting less overall agreement (marginal *M* = 53.78, *SE* = 2.82) than their defusion group counterparts (marginal *M* = 65.73, *SE* = 2.65). The two groups did not differ from each other at pretreatment, *t*(53) = 1.94, *p* = .057, but did so at posttreatment, *t*(53) = 2.98, *p* = .004, with a medium effect size (*d* = 0.52).

The prevalence of the ergodic fallacy within psychological research, or the unsupported assumption that aggregate comparisons between groups are reflective of individuals within them, has received increasing attention as of late (Speelman et al., 2024). Accordingly, we conducted a pervasiveness analysis to determine if our overall findings of equivalence between the two interventions may have obscured possible differences in their impact at the level of individuals (e.g., Speelman & McGann, 2020). We found no evidence of this, however. The two groups did not differ on any of the six ratings in the proportion of participants displaying improvement versus no change/deterioration, or in their levels of improvement (≤49 points vs. >50 points), suggesting that individuals had a similar distribution of reactions to the two interventions.

## 4. Discussion

The major purpose of this project was to compare the impact of extending defusion exercises common to ACT to metacognitive restructuring components integral to MCT in addressing meta-worries that typify GAD. With two exceptions, the two interventions were found to be indistinguishable in significantly reducing believability in and distress associated with targeted worries as well as other meta-worries that were not specifically addressed. Metacognitive defusion and metacognitive restructuring were also equivalent in increasing acceptance of both targeted and untargeted worries, albeit with a more modest effect size concerning the latter. Moreover, our pervasiveness analysis suggests that these findings at the aggregate level did not obscure any individual variability in responsivity to the two interventions.

One exception to simply and unconditionally concluding equivalence between the two conditions concerns a main effect for believability of targeted worries favoring metacognitive restructuring, while a second involves the significantly shorter average presentation time for this intervention. There was no interaction between pre- to posttreatment reductions in the endorsement of targeted worries and treatment conditions. However, the marginal mean of believability ratings when collapsed across the two assessment occasions was significantly lower for the restructuring group. This finding is attributable to a significant difference with the defusion condition at posttreatment in the absence of any similar difference between the two at pretreatment. In effect, change scores in the aggregate for participants in the restructuring group were not significantly greater than their counterparts even though their continued endorsement of the targeted worry was significantly lower at posttreatment.

Further consideration of this seemingly slight advantage for metacognitive restructuring should be undertaken within the context of differences in session duration. Metacognitive restructuring attained the same, if not even more preferable effects, as metacognitive defusion despite being presented on average for a significantly, but relatively, shorter period of time (i.e., 3.5 min), suggesting that metacognitive restructuring may be a more efficient intervention. It is unclear how to account for the difference in the duration of sessions. The doctoral students who delivered the two interventions in effect were free to continue the sessions until they judged that participants were unlikely to benefit from extending them any longer, provided the 30 min limit was met. Insofar as the impact of the two interventions was comparable, it would seem to follow that the duration of sessions were longer for metacognitive defusion quite possibly because the student therapists correctly ascertained that more of it was needed to attain the same effects as metacognitive restructuring, especially since participants undoubtedly were more accustomed to logically disputing beliefs than in using defusion exercises. In retrospect, another interpretation, however, would also seem to warrant some consideration. The significantly longer presentations of metacognitive defusion on average may have been more attributable to the student therapists, as mentioned, being more acquainted with ACT than MCT than the relative responsivity of participants to the two interventions. As a consequence, they quite possibly felt less comfortable in presenting metacognitive restructuring given their lesser familiarity with it, and were able to limit their unease by ending those sessions earlier.

In any event, further research would be required to more clearly determine whether metacognitive restructuring enjoys a more apparently favorable cost–benefit ratio than metacognitive defusion. This could be undertaken in at least two ways. One option would be to predetermine the length of all sessions (e.g., 15 min with a sample comparable to ours) rather than allowing them to vary, as was the case in this project, to ensure that an equivalent “dose” of each treatment is administered. The other possibility would be to use a trials-to-criterion type of design preferably with clinical samples as the logical next step in comparing the efficiency of these kernels. Sessions could continue until some predetermined goal, such as a specified reduction in believability in target worries occurs. In such a preparation, session duration becomes the independent variable, and unlike what may have occurred in this study, the criterion for discontinuation is objectively defined rather than being left up to the judgment of graduate student therapists.

A secondary albeit more exploratory purpose of this project, but one nonetheless consistent with the agenda of PB-CBT, was to examine if overall and/or differential responsivity to the two interventions was moderated by levels of clinically relevant variables. Out of a large domain of options, we chose measures of generalized anxiety, dispositional worrying, and depression to evaluate as possible moderators, with no evidence that any of the three served this function. This was despite, but perhaps also attributable to, elevated levels of each. To the extent that our sample could be regarded as a subclinical one, the high levels for all three ostensible moderating variables may have essentially created a ceiling effect with insufficient variability in each. Limited variability in the dependent variables as well as underpowered analyses in light of our modest sample size may have also been contributing factors. In addition, the possibility that simply other nondemographic variables than the ones that we selected, such as measures of self-compassion, mindfulness, or other processes contributory to psychological flexibility, might moderate responsivity to the two interventions seems worthy of further consideration and investigation with larger samples.

While we believe our overall findings have potentially relevant implications for extending a PB-CBT approach to treatment of GAD, further research that particularly addresses acknowledged shortcomings of this project would seem necessary for this possibility to be realized. Most obvious are limitations to both its internal and external validity. Like the earlier work of Deacon et al. (2011) in comparing defusion and cognitive restructuring with negative self-referential thoughts, we also opted not to include a control condition for at least two reasons. Given the status of MCT as an evidence-based approach for treatment of GAD, we largely viewed metacognitive restructuring as a “benchmark” against which the impact of metacognitive defusion could be evaluated, thus minimizing the need to include a control condition against which both interventions could be compared. Our other reason for omitting a control group was more pragmatic in nature. We went to considerable effort over a 2-year period of time to select a rather modest subclinical sample (*N* = 55) and opted not to further underpower our analyses by randomly assigning them to a third condition. Nonetheless, further research would be required to rule out the possibility that the equivalent impact seen for our two interventions might be attributable to nonspecific or placebo effects.

The major threat to the external validity of this project is not unique to it but endemic to all analog laboratory-based studies. While it appears that our final sample could be regarded as a subclinical one given their elevated levels of generalized anxiety, dispositional worrying, and comorbid depression, they did not actively self-initiate treatment. Accordingly, it remains unclear whether our overall findings can be generalized to those who present themselves for outpatient treatment of GAD. It seems reasonable to expect that more extensive presentations of both interventions would be necessary to attain effects comparable to ours, but this itself is an empirical question that could be examined by analyzing dose–response curves.

PB-CBT recognizes the importance of identifying both moderating and mediating variables of responsivity to therapeutic kernels. Insofar as a project thoroughly investigating dose–response curves would include multiple sessions of each treatment, an opportunity not afforded to us would be available in which to repeatedly collect and analyze process measures as possible mediating variables. Certainly, measures apart from the three we administered at pre and posttreatment as dependent variables for both the targeted and untargeted worries could be considered as mediating variables, but more closely examining changes in the relationship among ratings of believability, distress, and willingness over time might reasonably take precedence. Such analyses would of necessity require designating which rating(s) should be regarded as dependent variables and which one(s) ought to be considered as potentially moderating variables. For example, if lower distress is the desired outcome, are reductions in believability, increases in acceptance as assessed by willingness ratings, or both mediators of it? Moreover, are any mediational processes themselves moderated by type of intervention, for instance, such that reduced distress is mediated by reduced believability with metacognitive restructuring, but by increased willingness with metacognitive defusion? Alternatively, decreased believability rather than distress could be construed as the dependent variable with increased acceptance still considered as the mediating variable for one or both treatments.

To the degree that comparing the impact of isolated treatment elements, such as metacognitive defusion and metacognitive restructuring, to determine if each are potential therapeutic options may be a necessary first step within the PB-CBT approach, the overall results of this project in our view can be seen as making a modest albeit limited contribution in assisting those who struggle with GAD. To realize the full agenda of this approach, however, such investigations must be complemented by moderating and mediational research that is sensitive to clinically relevant outcomes occurring at the level of individual clients. The findings of such projects could assist clinical decision making by determining if both metacognitive restructuring and defusion overall represent viable options in treatment of Type 2 worrying in GAD, the specific circumstances and conditions in which one might be preferred over the other, and their respective mechanisms of action.

## Figures and Tables

**Table 1:** Participant demographic characteristics and descriptive statistics of putative moderating variables.

Group	Metacognitive Restructuring (*N* = 26)	Metacognitive Defusion (*N* = 29)	Total (*N* = 55)
Age (SD)	21.2 (6.4)	19.2 (1.3)	20.1 (4.5)
Birth Gender			
Male	5	10	15
Female	21	19	40
Gender Identity			
Cisgender Male	5	8	13
Cisgender Female	21	19	40
Transgender Female	0	1	1
Fluid	0	1	1
Ethnicity			
Latine	5	6	11
Non-Latine	21	23	44
Race			
White	19	19	38
Asian	2	5	7
Black	4	2	6
Native American	1	3	4
MCQ-30 (SD)			
Neg. Agreement ^a^	21.88 (3.88)	23.76 (4.48)	22.87 (4.27)
Neg. Distress ^b^	19.46 (3.58)	22.27 (4.93)	20.94 (4.53)
GAD-7 (SD)	10.19 (3.91)	10.38 (5.23)	10.29 (4.61)
PSWQ (SD)	62.00 (10.92)	63.07 (11.41)	62.56 (11.09)
BDI-II (SD)	17.27 (9.23)	19.34 (10.59)	18.36 (9.93)

^a^ Level of agreement with negative beliefs about worry. ^b^ Level of distress caused by negative beliefs about worry. Significantly higher level of distress (*p* = .02) for metacognitive defusion group.

**Table 2:** Ratings of believability, distress, and willingness for meta-worries at pre- and posttreatment.

Group	Metacognitive Restructuring (*N* = 26)		Metacognitive Defusion (*N* = 29)		Total (*N* = 55)	
	Pre	Post	Pre	Post	Pre	Post
Targeted Worry						
Believability ^1^	75.00 (13.63)	32.31 (20.26)	82.41 (14.55)	48.96 (21.10)	78.91 (14.49)	41.09 (22.17)
Distress ^1^	71.54 (15.15)	28.08 (21.73)	76.21 (13.73)	36.55 (23.49)	74.00 (14.48)	32.54 (22.87)
Willingness ^2^	34.23 (25.32)	56.54 (34.05)	39.65 (18.80)	53.10 (30.36)	37.09 (22.08)	54.73 (31.91)
Untargeted Worries						
Believability	51.77 (15.31)	32.31 (20.16)	59.38 (17.01)	39.17 (20.25)	55.78 (16.53)	35.93 (20.31)
Distress	47.62 (18.30)	26.38 (18.73)	52.90 (16.30)	29.72 (18.19)	50.40 (17.32)	28.14 (18.35)
Willingness	46.46 (26.39)	60.08 (33.35)	50.79 (24.98)	59.38 (33.16)	48.73 (25.51)	59.71 (32.94)

*Note*. Parenthetical data are standard deviations. ^1^ Decreased pre-to-post scores reflect improvement. ^2^ Increased pre-to-post scores reflect improvement.

**Table 3:** Summary of analyses of variance of ratings of believability, distress, and willingness for meta-worries.

Effect	Assessment Occasion			Treatment Condition			Interaction		
	*F* ratio	*p*	η^2^p	*F* ratio	*p*	η^2^p	*F* ratio	*p*	η^2^p
Targeted Worry									
Believability ^a^	16.82	<.001	.252	8.50	.005	.145	0.46	.501	.009
Distress	145.55	<.001	.733	2.98	.090	.053	0.30	.583	.006
Willingness	37.88	<.001	.415	0.02	.885	.000	2.31	.135	.042
Untargeted Worries									
Believability	103.35	<.001	.661	2.53	.118	.046	0.04	.849	.001
Distress	116.23	<.001	.687	0.97	.328	.018	0.22	.639	.004
Willingness ^a^	10.54	.002	.174	0.90	.346	.018	0.10	.750	.002

*Note.* For ANOVAS, *df* = 1, 53. ^a^ Results of ANCOVA with session duration as the covariate; *df* = 1, 50.

## Data Availability

The raw data supporting the conclusions of this article will be made available by the authors on request.

## Note

1 Copies of the protocols are available upon request by contacting the corresponding author.
